# Molecular Genetic Analysis of Perioperative Colonization by Infection-Related Microorganisms in Patients Receiving Intraoral Microvascular Grafts

**DOI:** 10.3390/jcm13144103

**Published:** 2024-07-13

**Authors:** Henriette Louise Moellmann, Katharina Kommer, Nadia Karnatz, Klaus Pfeffer, Birgit Henrich, Majeed Rana

**Affiliations:** 1 Department of Cranio-and-Maxillo Facial Surgery, University Hospital Düsseldorf, Moorenstraße 5, 40225 Düsseldorf, Germany; nadia.karnatz@med.uni-duesseldorf.de (N.K.); rana@med.uni-duesseldorf.de (M.R.); 2 Institute of Medical Microbiology and Hospital Hygiene, Heinrich Heine University Düsseldorf, 40225 Düsseldorf, Germany; katharina.kommer@rwth-aachen.de (K.K.); klaus.pfeffer@hhu.de (K.P.); birgit.henrich@hhu.de (B.H.)

**Keywords:** oral microbiome, microvascular reconstruction, antibiotics

## Abstract

**Background/Objectives**: In oral and maxillofacial surgery, the reconstruction of defects often involves the transfer of skin tissue into the oral cavity utilizing microvascular grafts. This study investigates postoperative changes in microbial colonization following intraoral microvascular transplantation, as well as potential influencing factors. **Methods**: In 37 patients undergoing intraoral reconstructions, pre- and postoperative swabs were taken from the donor and recipient regions to quantify the seven selected marker bacteria using TaqMan PCRs. Patient-specific factors and clinical data were also recorded. **Results**: The infection-associated *Acinetobacter baumannii* tended to decrease postoperatively, while the infectious pathogens *Pseudomonas aeruginosa*, *Enterococcus faecalis* and the family of *Enterobacteriaceae* showed a postoperative increase without being directly associated with a clinical infection. *Streptococcus mitis* showed a significant postoperative decrease on buccal mucosa and increase on the graft surface (oral dysbiosis) and was significantly reduced or displaced by other bacteria (e.g., *Mycoplasma salivarium*, positive selection) when treated with ampicillin/sulbactam. **Conclusions:** The cutaneous microbiome of the graft adapts to the local intraoral environment. Postoperative shifts in oral bacterial colonization and an increase in infection-relevant bacteria were observed. These perioperative changes in colonization are also influenced by the administration of ampicillin/sulbactam. Consequently, single doses of antibiotics appear to be more beneficial compared to longer-term preventive use.

## 1. Introduction

In oral and maxillofacial surgery, extensive tissue defects are often associated with severe loss of function and have esthetically disfiguring and stigmatizing effects [1,2,3,4,5,6]. If primary wound closure is not possible, reconstruction using microvascular grafts is the gold standard [3,4,7,8,9]. The anterolateral thigh (ALT), radial forearm, and latissimus dorsi muscle or myocutaneous flaps are the most commonly used donor sites [10].

Alternative flap donor sites such as the lateral arm or the medial sural artery perforator (MSAP) flap are also being used more and more frequently. They offer a better color match and less morbidity at the donor site and can be an alternative in cases of previous flap loss, recurrence or the need for multiple free flaps [11,12,13]. If bone reconstruction is required in addition to soft tissue reconstruction, virtual surgical planning (VSP) is used for complex cases [14,15,16,17]. The perioperative changes in microbial colonization of the surgical site and the oral cavity following intraoral microvascular grafts have been described to a limited extent in the literature. It has been demonstrated that an altered nutrient availability, oxygen concentration, host response or an altered pH value can lead to dysbiosis due to the proliferation of opportunistic bacteria [18,19]. It seems obvious that highly invasive procedures such as intraoral microvascular reconstructions lead to a distinct change in the oral milieu. Other factors that also indicate a disruption in the homeostatic balance between the host and the oral microflora include the following: difficult oral hygiene due to swelling or pain [20], long-term hospitalization [21], the use of antimicrobial mouth rinses [22,23], postoperative antibiotics [24], potential postoperative xerostomia [25], and the transfer of extraoral tissue into the oral cavity [26]. Durand and Eckert et al. were able to show that wound infections following oral microvascular transplantation are often triggered by pathogens that are not commonly part of the healthy oral flora [27,28]. The extent to which foreign pathogens can colonize and multiply in the oral cavity under certain conditions is currently being investigated [29,30,31]. The following factors have already been identified as possible influences on intraoral microbial colonization and the development of oral dysbiosis: long retention times (increased *E. faecalis* and *E. faecium*, as well as various representatives of the *Enterobacteriaceae*) [21]), nosocomial infections (increased *S. aureus, A. baumannii, P. aeruginosa* and various representatives of the *Enterobacteriaceae*) [32,33,34], intraoral reconstruction [27,28,35,36,37], and the severity of the disease (pharyngeal colonization of Gram-negative bacilli) [38].

Intraoral tumor diseases [39,40,41,42,43] and their surgical resection [44] have a significant impact on oral microbial colonization. Chan et al. (2021) investigated the postoperative long-term changes in the oral microbiome using next-generation sequencing. They observed a postoperative decrease (6 months) in some periodontopathogenic genera such as *Fusobacterium*, *Capnocytophaga*, *Pophyromonas, Leptotrichia*, *Aggregatibacter* and *Treponema*, while the commensals of the oral flora *Streptococcus* and *Rothia* increased. In addition, the authors showed a correlation between the postoperative shifts in the oral microflora and the patient-specific prognosis: the 3-year survival rate improved with a reduced relative frequency of the periodontopathogenic genera *Capnocytophaga*, *Prevotella* and *Leptotrichia* and with an increased relative frequency of the two commensal genera *Streptococcus* and *Rothia* six months postoperatively [44]. Dental and periodontal health also have an impact on the development of postoperative infections in the oral cavity. Sato et al. showed that professional dental cleaning and oral hygiene instructions significantly reduced the risk of wound infections after excision of oral squamous cell carcinoma [45]. Usubuchi et al. demonstrated that preoperative dental treatment (scaling, treatment of deep carious lesions and severe periodontitis) reduced the risk of postoperative infection after microvascular grafts in the head and neck region [46]. In addition, the activity of the immune system appears to change after extensive oral surgery. This was shown by Heimlich et al. with a significant drop in the total lymphocyte count, as well as CD4+ and CD8+ T lymphocytes after long operations lasting 7 h or more [47]. Kageyama et al. (2020) showed an influence of the surface composition of the oral epithelia on microbial colonization. They hypothesized a connection between the composition of the salivary microbiome and a postoperatively reduced tongue surface area. The authors used 16S rRNA gene sequencing and qPCR analysis to investigate the microbiome of stimulated saliva samples from patients with tumor resections in the tongue area. The analysis showed a significant postoperative decrease in microorganisms that are the predominant inhabitants of the dorsum of the tongue (*S. salivarius*, *P. melaninogenica*, *P. histicola and Actinomyces* spp.). In contrast, dental plaque bacteria such as *L. mirabilis*, *N. flava*, *S. sanguinis* and *F. nucleatum* increased significantly. This reflected a shift in the salivary microbiome in favor of plaque-associated bacteria (resident inhabitants of the oral flora) as part of the reduction in the tongue surface caused by the tumor resection. Only two of the patients included in the study underwent defect reconstruction by means of microvascular transplantation. The authors pointed out the need for further research in larger sample sizes in order to investigate the effect of microvascular grafts on oral microbial colonization, as an influence of the epithelial characteristics of the outer skin, which differ from the oral mucosa, on microbial colonization is obvious [48].

The aim of this study was to detect perioperative microbiological changes in patients following intraoral reconstructions using microvascular grafts. Other factors influencing microbial colonization, such as postoperative xerostomia, antibiotics administered, and the state of oral hygiene and oral health were also evaluated.

## 2. Materials and Methods

### 2.1. Study Population and Samples

Patients from the Department of Oral and Maxillofacial Plastic Surgery at the University Hospital Düsseldorf were included in the study if surgery was indicated for intraoral tissue defect reconstruction using microvascular transplantation. This study was approved by the local ethics committee at the University of Düsseldorf, Germany (Approval number 2021-1342). Alongside demographic data (age, gender, body mass index, and tobacco consumption), clinical parameters (such as laboratory tests and ASA classification) and patient-specific factors (including defect type, defect localization, type of graft, perioperative body temperature, and administration of antibiotics) were recorded. Preoperative swabs were collected from the cheek (W0) and the extraoral donor region (T0), while postoperative swabs were taken 1–3 days (cheek (W1), graft (T1), and intraoral suture (N1)) and 6–9 days (cheek (W2), graft (T2), and intraoral suture (N2)) after surgery. Cheek swabs were consistently obtained from the intraoral buccal mucosa on the contralateral side of the defect. The preoperative cheek swab (W0) served as a reference sample.

### 2.2. Genomic DNA Preparation and TaqMan Polymerase Chain Reaction (PCR)

Material attached to the swabs was resuspended by vortexing in 200 μL G2 storage buffer. The suspension was supplemented with 12.5 μL Proteinase K solution (1 mg/mL Proteinase K) and incubated for 30 min at 56 °C. (G2 storage buffer and Proteinase K were sourced from EZ1 DNA Tissue Kits, QIAGEN GmbH, Hilden, Germany). Total genomic DNA isolation was performed by a semiautomatic DNA preparation using BioRobot EZ1 and EZ1 DNA Tissue Kit according to the manufacturer’s instructions (protocol “Bact_200 µL”) with an elution volume of 50 μL. The eluate was stored at −20 °C until further use.

In-house TaqMan PCRs to quantify the selected pathogens, total eubacterial load and human GAPDH were carried out in a total volume of 25 μL consisting of 2× Takyon™ No Rox Probe MasterMix UNG (Eurogentec, Seraing, Belgium; containing Takyon™ DNA polymerase, 5.5 mM MgCl_2_, dNTPs (including dUTP), uracil-N-glycosylase and stabilizers), 300 nM each forward and reverse primer, 200 nM labeled probe, and 2.5 μL of template DNA. Amplicon-carrying plasmids were used as quantification standards in concentrations of 10^7^, 10^5^ and 10^4^ copies/μL for detection of the total bacterial load and *Enterobacteriaceae* and 10^7^, 10^5^ and 10^2^ copies/μL for the other bacterial species investigated and human GAPDH. Thermal cycling conditions were as follows: 1 cycle at 95 °C for 10min followed by 45 cycles at 95 °C for 15 s, and 60 °C for 1 min. CFX96 Real-Time Systems (BioRad, Shinagawa City, Tokyo, Japan) devices were used for all TaqMan PCRs performed. Data were analyzed using the BioRad CFX Manager 3.1 software.

### 2.3. Data Normalization

After TaqMan PCR analysis, the detected copies/PCR were converted into the number of genome equivalents (GE) per sample. To further normalize the results, numbers of total eubacterial loads and human cells removed from the swab were determined. The results were normalized by dividing the respective genome equivalents per sample by the corresponding total eubacterial load or human GAPDH. Thus, three normalized values were available for each species-specific PCR: the genome equivalents of the respective species (1) per sample, (2) per total eubacterial load and (3) per human GAPDH. A result was considered statistically significant in this study if two of the three data sets yielded statistically significant results.

### 2.4. Statistical Analysis

The minimum required sample size was determined using the G*Power 3.1 software. An analysis of variance (ANOVA) was used as a statistical test for the sample calculation for linked samples, as it was initially unclear whether the measured values were normally distributed. With an estimated effect size of 0.29, a test power of 95% and a significance level of 5%, the minimum number of study participants was 33. The statistical calculations were carried out using SPSS software version 28.0.0.0. A normal distribution of the measured values was tested using the Shapiro–Wilk test [49]. The Wilcoxon test was used for the comparative analysis of two metric measurements [50] The Friedman test was used for comparative analyses of more than two metric measurements [51]. The Spearman correlation was used for correlation analyses of ordinal-scale indices with metric measurement data (microbial colonization) [52]. The Mann–Whitney U test was used for the comparative analysis of two independent samples [52]. The Kruskal–Wallis test was used for comparative analyses of more than two independent samples [53].

## 3. Results

The 37 patients included in the study were characterized according to the following parameters (Table 1):

### 3.1. Antibiotics

On the day of surgery, all study patients received an antibiotic in the form of intraoperative “single-shot” antibiotics, with n = 29 patients (78.4%) receiving intraoperative antibiotics with ampicillin/sulbactam and n = 8 patients (21.6%) with clindamycin due to penicillin allergy. Pre- and postoperative antibiotic medications varied.

Six patients received a single dose of 2 g ceftriaxone or 500 mg ciprofloxacin between the sixth and second preoperative day immediately prior to percutaneous endoscopic gastrostomy (PEG). Postoperatively, n = 15 patients (40.5%) received no further antibiotic treatment during the study period, while n = 15 patients (40.5%) received at least one more day of ampicillin/sulbactam and n = 6 patients (16.2%) received at least one more day of clindamycin. In individual cases, amoxicillin/clavulanic acid or piperacillin/tazobactam were used postoperatively.

### 3.2. Molecular Genetic Detections

**Figure 1:**
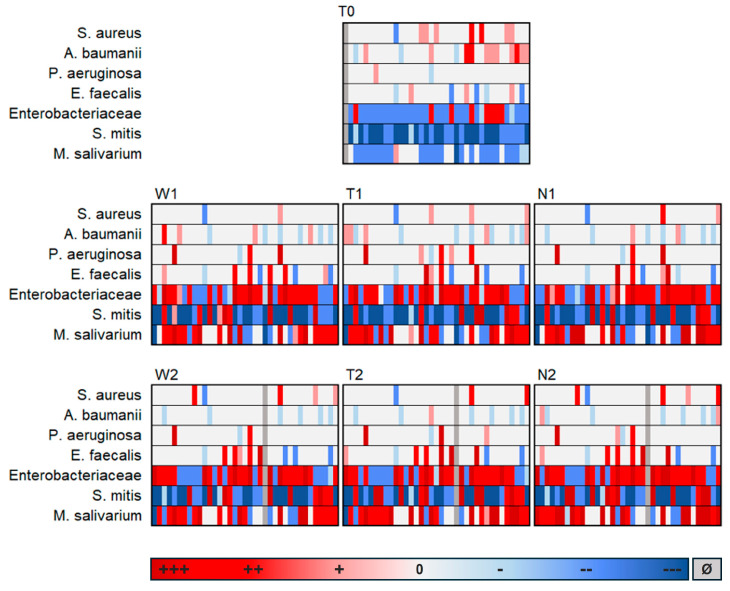
Difference in the respective load of bacteria detection in relation to the reference swab W0; Shown are the swabs from the cheek (W), graft (T) and suture (N) at the preoperative time point (0), early postoperative time point (1) and late postoperative time point (2), +++ = Highly increased bacterial load (Δ [X] − [W0] > 1 × 10^6^ GE/sample); ++ = Moderately increased bacterial load (Δ [X] − [W0] > 1 × 10^3^ and < 1 × 10^6^ GE/sample); + = Slightly increased bacterial load (Δ [X] − [W0] > 0 and < 1 × 10^3^ GE/sample); 0 = No positive evidence; - = Slightly reduced bacterial load (Δ [X] − [W0] > −1 × 10^3^ and <0 to GE/sample), -- = Moderately reduced bacterial load (Δ [X] − [W0] > −1 × 10^6^ and < −1 × 10^3^ GE/sample); --- Highly reduced bacterial load (Δ [X] − [W0] > −1 × 10^6^ GE/sample); Ø = No data available.

Following TaqMan PCRs, the quantitative detection of the bacteria analyzed per swab was shown based on the data of all 37 study participants. The detected pathogen loads were to be analyzed in relation to a selected reference. As the focus of the comparative analysis was on intraoral perioperative changes in pathogen detection, the preoperative buccal swab W0 was selected as the reference swab. Figure 1 shows the difference between the detected bacterial load per swab and the corresponding reference swab W0 per patient:

It was revealed that the bacterial colonization examined on the preoperative graft surface (T0) differed fundamentally from all postoperative swabs, whereas the postoperative swabs (regardless of localization or postoperative time) hardly differed from each other. Therefore, it can be concluded that the composition of the analyzed species on the dermal graft after transfer to the intraoral environment resembled that of the buccal mucosa. This is remarkable considering the different surface characteristics of the oral mucosa and the external skin. Additionally, there appeared to be surprisingly little influence in the opposite direction, meaning hardly any impact of the transplanted external skin on the oral composition of the analyzed species.

It was also evident that *S. mitis* was reduced in the postoperative course compared to W0, while *M. salivarium* increased. *Enterobacteriaceae*, *E. faecalis* and *P. aeruginosa* tended to increase postoperatively, while *A. baumannii* tended to decrease.

**Figure 2:**
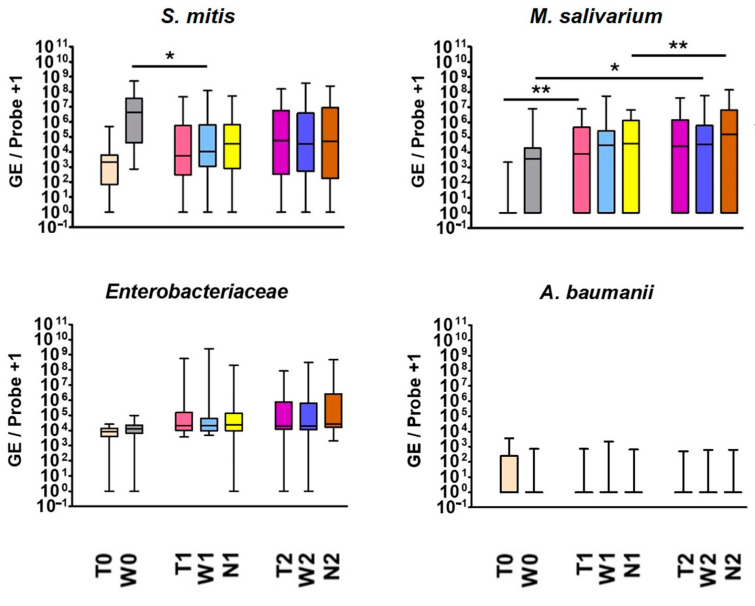
Exemplary results of quantitative bacterial detections (GE/sample) of *S. mitis*, *M. salivarium*, *Enterobacteriaceae* and *A. baumannii*; the results of all 37 participants are shown on cheek (W), graft (T) and suture swab (N) at the preoperative time point (0), early postoperative time point (1) and late postoperative time point (2). * *p* <0.05; ** *p* <0.01.

Figure 2 shows the exemplary results of *S. mitis*, *M. salivarium*, *Enterobacteriaceae* and *A. baumannii* over time:

Positive detections of *S. mitis*, *M. salivarium* and *Enterobacteriaceae* occurred regularly. A trend towards an increase in *Enterobacteriaceae* was observed during the perioperative course, while *A. baumannii* tended to decrease postoperatively, especially on the graft surface.

While *M. salivarium* was hardly detectable in the (dermal) donor region, it rose significantly on the graft postoperatively (*p* = 0.005/0.007/0.015). Consequently, the graft was colonized by *M. salivarium*, which is typically found in the oral cavity, after being transferred intraorally. However, the microorganism also increased in the purely intraorally localized examined regions during this study, which suggests that additional factors such as positive selection by administered beta-lactam antibiotics favored a postoperative increase. On the cheek, the microorganism increased significantly from preoperative to late postoperative time point (*p* = 0.203/0.024/0.005). Additionally, there was a significant increase on the suture in the postoperative course (*p* = 0.002/0.412/<0.001).

*S. mitis* exhibited a gradual increase on the graft surface until the colonization by this typical oral microorganism ultimately matched that of the cheek mucosa. The microorganism behaved oppositely when considering cheek swabs separately: It significantly decreased from preoperative to postoperative time point 1 (*p* = 0.01/0.034/<0.001). The postoperative reduction in *S. mitis*, a typical representative of healthy oral flora, may indicate postoperative dysbiosis.

### 3.3. Culture-Based Detections and Resistances

In addition to the molecular genetic analysis carried out as part of the current study, seven study patients underwent postoperative culture and resistance testing, which was commissioned by the Clinic for Oral and Maxillofacial Plastic Surgery for diagnostic purposes. The following table (Table 2) shows the detected species and respective resistances:

Although the samples for cultural detection were all taken extraorally and therefore the localization at no point corresponded to the samples for molecular genetic detection (only taken intraorally postoperatively), the cultural detections largely matched the molecular genetic detections: All positive culture results for *K. aerogenes*, *E. coli*, *E. faecalis* and *P. aeruginosa* were consistent with the results of the molecular genetic analysis. Only *K. oxytoca* and *E. cloacae* detected in the culture were not reliably reproduced in the molecular genetic analysis, which may be either due to the low sensitivity of the *Enterobacteriaceae* TaqMan PCR or the differing swab location. Positive detections of *P. vulgaris* were not included in the molecular genetic analysis.

### 3.4. Wound Complications and Inflammation Parameters

Three patients experienced graft complications in the postoperative course. While the microvascular graft had to be subsequently removed in one patient due to a thromboembolic complication, the graft was retained in two patients despite wound-healing disorders. In one patient, a wound dehiscence developed in the extraoral area of the graft during the postoperative course, which was subsequently covered by local flap plasty. The graft healed without irritation in the intraoral area. Although no signs of infection were clinically recognizable in the surgical area, molecular genetic analysis showed a strong postoperative increase in *Enterobacteriaceae*, which was present at high levels (>1 × 10^6^ GE/sample) in five of the six postoperative swabs. *S. aureus* was detected in this patient almost throughout the entire course of the study. The molecular genetic evidence of *Enterobacteriaceae* and *S. aureus* corresponded with the results of the culture procedure carried out in parallel, which, in addition to positive evidence of *P. vulgaris*, also provided evidence of *E. coli* and *S. aureus.* All three species exhibited (in some cases multiple) antibiotic resistance (see Table 2).

In another patient, a clinically detectable infection (redness, swelling) developed in the postoperative course in the intraoral suture area. At the molecular genetic level, high levels of infection-relevant *Enterobacteriaceae*, as well as moderate or high levels of *E. faecalis* and *P. aeruginosa*, were detected in the current study. For this patient, no additional culture procedure was conducted.

In the postoperative course, 62% of patients had no fever, while 30% developed mild fever (38.0–38.5 °C) and 9% moderate-to-high fever (>38.5 °C). Mild fever mostly occurred within the first 2 days postoperatively. Moderate or high fever occurred exclusively within the first 2 days. A postoperative increase in body temperature was not statistically significant. Furthermore, no statistically significant correlation was found between the perioperative body temperature and the detection of the bacteria analyzed, nor between febrile temperatures and patient demographics or antibiotics administered.

**Figure 3:**
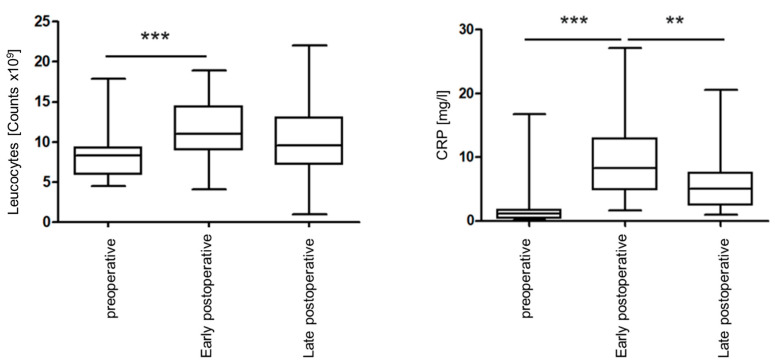
Perioperative leukocyte counts (**left**) and perioperative C-reactive protein values (**right**) of all study participants. ** *p* <0.01; *** *p* <0.001.

The following boxplots (Figure 3) show the perioperative leucocyte counts and plasma levels of C-reactive protein for all 37 study participants:

Both leukocyte count and CRP values were elevated early postoperatively and recovered by the end of the postoperative phase. A statistically significant increase in the leukocyte counts from the preoperative to the early postoperative time point (*p* < 0.001) was observed with a slighter non-significant decrease later (*p* = 0.093). CRP values also showed a significant increase early postoperatively (*p* < 0.001), with a significant decrease later (*p* = 0.003). No statistically significant correlations were found between the perioperative detection of the microorganisms analyzed and the perioperative leukocyte counts, or CRP values determined. The duration of a postoperative leukocytosis or CRP increase was not significantly related to bacterial detection. Postoperative increases in CRP and leucocyte counts were associated with clinical signs of infection in individual cases. Both cases of wound complications showed early postoperative highly elevated CRP values, the highest in the study.

In summary, postoperative increases in pathogens only actually triggered a wound infection in individual cases. Similarly, increases in CRP, leukocyte count, and body temperature did not indicate wound infections. Overall, there were no significant correlations between inflammatory parameters and the detected perioperative bacterial colonization.

## 4. Discussion

### 4.1. Selection of the Microorganisms

The selection of pathogens was based on the current literature [27,28,36,37,54,55,56,57]. In the context of a wound infection that occurred in this study, *E. faecalis*, *Enterobacteriaceae* and *P. aeruginosa* were detected in all postoperative swabs in moderate or high loads. In accordance with the literature, this was therefore a mixed infection carried by classical nosocomial bacteria. However, it should be noted that the loads of the species detected for this patient only made up a small proportion of the total eubacterial load of the respective swabs. It can therefore be assumed that other microorganisms that were not detected may have been involved in the infection.

As a representative of healthy oral flora, the species *S. mitis* was selected, which is strongly associated with oral health in the literature. Some of the beneficial effects on oral health, such as the protection of the periodontium from tissue damage and the inhibition of pathogens, have been described [58,59,60,61,62]. It has been shown that the microorganism can be reduced in dysbiotic conditions and in association with diseases in the oral cavity and human body [62,63,64,65]. Therefore, a loss or decrease in the *S. mitis* load from the oral flora was considered a sign of dysbiosis in this study.

Furthermore, *M. salivarium* was included in the selection of detected microorganisms, which is generally considered a commensal of the oral flora, although there have also been occasional reports of associations with pathological processes [66,67,68,69,70,71,72,73]. *M. salivarium* was particularly well-suited for investigating the effect of antibiotic-associated dysbiosis, as *M. salivarium*, being a cell wall-less bacterium, does not respond to cell wall-acting and frequently administered beta-lactam antibiotics [74,75].

### 4.2. Patient Characteristics

The study patients were 51% female and 49% male. According to the literature, men are affected by oral cancer 1.5 times more frequently and two to three years earlier than women due to a higher exposure to risk factors [76,77]. On average, the study patients were 65.3 years old, which is in line with the average age in comparable studies [2,78]. The patients had an average BMI of 25.4, while slightly lower BMIs of between 21.6 and 24.6 were reported in comparable studies [2,37,46]. According to Bartella et al. (2018), an increased BMI is associated with an increased risk of postoperative infections after head and neck surgery [37], while according to Kruse et al. (2010), BMI has no influence on the survival of microvascular transplants in the head and neck region [79]. The ASA score I or II (62%) is in line with ASA scores of patients in comparable studies [2,55]. The literature provides evidence that an increased ASA score significantly increases the infection and mortality rates of microvascular grafts in the head and neck region [2,79]. In the current study, no correlation was found between postoperative graft survival or infections and the ASA score.

### 4.3. Complications and Inflammation

Postoperative infection occurred in one of the 37 patients and wound dehiscence in the extraoral area of the graft in another patient. This wound infection and dehiscence rate of 3% each is significantly lower than the values of 13.3 to 40.6% for infections described in the literature [2,27,36,78,80] and 14 to 29% for dehiscences [7,8]. This may be due to the overlap in sample collection from April 2021 to January 2023 with the additional hygiene protection measures introduced as part of the COVID-19 pandemic. A graft loss rate of 3% is slightly below the rates reported in the literature (5 to 8%) [8,81,82].

### 4.4. Perioperative Bacterial Colonization

The significantly reduced postoperative detection of *S. mitis*, which represents oral health, alongside increases in infectious pathogens like *P. aeruginosa*, *E. faecalis*, *Enterobacteriaceae* as well as *M. salivarium*, which is rarely associated with infection, strongly indicates postoperative dysbiosis. Typical pathogens of postoperative infections after intraoral microvascular transplantations are not part of the resident oral flora [27,28]. The microbiome of the external skin differs fundamentally from the oral microbiome [26]. The interactions of bacteria and body surfaces are based on the highly specific binding of adhesion molecules of the adhering microorganisms and certain receptors on the cell surface [42]. In the current study, there were hardly any differences between the postoperative colonization of the microorganisms examined on the cheek, suture, and graft. Very similar colonization patterns were found at the three localizations despite different epithelial and surface conditions. It was shown that *M. salivarium* can colonize the keratinized squamous epithelium to a similar extent as the buccal mucosa after the transfer of the extraoral skin surface into the mouth. The colonization of the graft was thus strongly influenced postoperatively by the surrounding oral flora, but conversely, the transfer of extraoral skin into the mouth had no significant influence on the oral colonization of the microorganisms examined. Consistent with this result, Kageyama et al. (2020) found no differences in oral microbial composition in patients who had received an oral microvascular graft compared to patients who had undergone primary closure of defects. Although the sample in this study was very small, the authors provided initial indications that the surface of the graft does not have as great an influence on the microbial colonization of the oral cavity, as might have been assumed [48]. One possible explanation for the postoperatively similar colonization patterns examined in the different localizations studied is the function of saliva as a fingerprint of the microbiome [83,84,85]. Although the microbial composition of saliva is not absolutely representative of the complete oral microbiome, saliva does contain microorganisms from various oral niches [19]. It is therefore conceivable that the saliva wetting the graft surface could transfer microorganisms from other oral niches such as the buccal mucosa to the graft.

### 4.5. Antibiotic Treatment

The bacteriostatic or bactericidal effect of an antibiotic inhibits sensitive species and chemically induced dysbiosis can occur. At the same time, non-sensitive species experience selection advantages, which can promote their proliferation [86,87,88].

The results showed that the microorganism of oral health *S. mitis* was widely represented in the preoperative buccal swab, whereas it had to give way to colonization by other microorganisms in the postoperative course. Longer-term administration of ampicillin/sulbactam for at least three days led to strong containment of the microorganism and drastic reductions in its proportion of the total bacterial count. Short-term antibiotics of ampicillin/sulbactam were also associated with significant reductions in the *S. mitis* load. Although medications with clindamycin also tended to reduce the bacterial load, this was less pronounced. Contrary to our results, viridans streptococci, which include *S. mitis*, can be inhibited similarly well by ampicillin/sulbactam and clindamycin according to the literature, although high resistance rates are reported in some cases [89]. In a large-scale review, Singh et al. (2022) examined the susceptibility rates of viridans streptococci to various antibiotics over the period from 2010 to 2020. The susceptibility rate of *S. mitis* to clindamycin was reported to be 83.8% on average over the period mentioned, while that to ampicillin was 81.0%, with no significant changes in susceptibility over the decade for either antibiotic [90]. While ampicillin/sulbactam medications of at least three days strongly reduced the colonization of *S. mitis* in our study, *M. salivarium* increased drastically; so, in some cases very high proportions of over 80% of the total bacterial load were achieved. The bacteria can therefore be regarded as antagonists after long-term administration of ampicillin/sulbactam. The fact that *M. salivarium* showed a strong increase after medication with ampicillin/sulbactam seems obvious, since this cell wall-less bacterium does not respond to the cell wall-active beta-lactam antibiotic [74,75,91]. Thus, a multiplication of the microorganism can be explained by a selection advantage over sensitive bacteria (such as staphylococci or streptococci like *S. mitis*).

Clindamycin exhibits limited efficacy against the entire *Enterobacteriaceae* family, while ampicillin/sulbactam and piperacillin/tazobactam are similarly ineffective against many members of this family. Additionally, *P. aeruginosa* is not effectively inhibited by either clindamycin or ampicillin/sulbactam. This lack of efficacy elucidates why, in the current study, increased detections of *Enterobacteriaceae* or *P. aeruginosa* were observed regardless of the antibiotic choice or duration of treatment. It can be inferred that the administered antibiotics further promoted postoperative increases in *P. aeruginosa* and representatives of *Enterobacteriaceae* by conferring a selective advantage over more sensitive species. Although antibiotic prophylaxis in head and neck surgery has been investigated in numerous studies, there are still controversial views regarding the choice and duration of antibiotic treatment. According to the literature, 30–80% of head and neck tumor surgery is covered with antibiotics, and antibiotic coverage can drastically reduce the risk of postoperative infection [54,92].

According to Durand et al. (2015), however, the choice of antibiotic for infection prophylaxis has a strong impact on the development of postoperative infections after microvascular transplants in the head and neck area: according to the authors, the risk of suffering a postoperative wound infection was more than doubled when clindamycin was used compared to ampicillin/sulbactam, cefazolin and other antibiotics. One of the reasons for this was the high incidence of Gram-negative rods after clindamycin medication. The authors identified clindamycin as a risk factor for the development of postoperative infections [27]. Other studies also reported an increased risk of postoperative wound infection after antibiotic prophylaxis with clindamycin [93,94]. Given the very low complication rates observed in our study, we are unable to determine the extent to which the choice or duration of antibiotic therapy influenced the development of postoperative wound infections. According to Bartella et al. (2018), the duration of antibiotic treatment did not appear to have any influence on the development of postoperative wound infections after oral surgery. Prolonged postoperative antibiotic administration as infection prophylaxis did not appear to offer any advantage over single-shot antibiotics administered perioperatively [37]. Vila et al. (2017) also described in their meta-analysis of 340 patients that a five-day postoperative antibiotic prophylaxis does not provide any advantage for infection prophylaxis compared to a one-day postoperative prophylaxis [92]. This is supported by the results of the current study: the fact that the oral colonization of the pathogens examined was not significantly reduced by prolonged antibiotic treatment compared to short-term antibiotic treatment suggests that prolonged postoperative antibiotic treatment does not offer the patient any advantage in terms of infection prophylaxis.

## 5. Conclusions

The colonization pattern of the examined bacteria on the postoperative graft surface adapts to the intraoral colonization. It can be assumed that the transplantation of extraoral skin surface into the oral cavity has a relatively minor influence on the development of postoperative dysbiosis following microvascular transplantation. However, the dysbiotic reduction in the *S. mitis* is significantly influenced by postoperative antibiotics like ampicillin/sulbactam. Therefore, standard antibiotics should be critically reviewed after microvascular transplantation in the oral cavity. It is recommended to exercise caution with the prolonged use of standard antibiotics following microvascular postoperative transplantation. This approach aims to minimize side effects, reduce the development of antibiotic resistance, prevent dysbiosis, and avoid the selection of non-sensitive and potentially infection-relevant species.

## Figures and Tables

**Table 1:** Overview of patient- and operation-specific factors.

Patient-Specific Factors	
Age	20 to 84 years; average age 65.2 years
Gender	Male: n = 18 (49%)
	Female: n = 19 (51%)
ASA-Score	ASA I: n = 5 (13.5%)
	ASA II: n = 18 (48.6%)
	ASA III: n = 12 (32.4%)
	ASA IV: n = 2 (5.4%)
BMI	14.5–37.7; average BMI at 25.4
Nicotine use	Smoking history:
	Yes: n = 24 (64.9%); No: n = 13 (35.1%)
	Smoking habit at the time of surgery:
	Yes: n = 15 (40.5%); No: n = 15 (59.4%)
	Pack years: Mean 31 PY; (2–98 PY)
Surgery-specific factors	
Type of graft	Radialis graft: n = 26 (71.1%)
	Fibula graft: n = 10 (26.3%)
	Anterolateral thigh (ALT) graft n = 1 (2.6%)
Localization of the defect	Lower jaw: n = 12 (32.4%)
	Upper jaw/palate: n = 7 (18.9%)
	Tongue: n = 5 (13.5%)
	Floor of mouth: n = 6 (16.2%)
	Lip: n = 1 (2.7%)
	Other/overarching localizations: n = 6 (16.2%)
Type of defect	Sole reconstruction (condition following tumor excision ^1^): n = 5 (13.5%)
	Squamous cell carcinoma: n = 26 (70.3%)
	Other tumor type ^2^: n = 2 (5.4%)
	Osteonecrosis of the jaw ^3^: n = 3 (8.1%)
	Keratocyst: n = 1 (2.7%)
Tumor stage	No tumor: n = 9 (24.3%)
	Stage 1: n = 6 (16.2%)
	Stage 2: n = 9 (24.3%)
	Stage 3: n = 6 (16.2%)
	Stage 4: n = 6 (16.2%)
	Recurrence: n = 1 (2.7%)
Postoperative graft complications	Intraoral wound infection: n = 1
	Wound dehiscence: n = 1 ^4^
	Complete graft loss: n = 1

^1^ Mucoepidermoid carcinoma n = 1, fibrosarcoma n = 1, squamous cell carcinoma n = 1, radiation-induced sarcoma n = 1 and adenocystic carcinoma n = 1; ^2^ adenocarcinoma n = 1; myoepithelial carcinoma n = 1; ^3^ MRONJ n = 2; osteoradionecrosis n = 1; ^4^ exclusively extraoral.

**Table 2:** Species and resistances detected in the cultivation process per patient; the type, localization, and time of sampling per patient are also shown.

Patient	Type/ Localization of the Sampling	Day (Postoperative)	Cultivation Evidence	Resistances
1	swab/ cervical	3	*K. aerogenes* ^1^ *E. coli* ^1^	S S
5	swab/ cervical	7	*P. aeruginosa* *E. cloaceae* ^1^	S S
11	Tissue/ infraorbital	1	*E. coli* ^1^	Ampicillin-R, Amoxicillin-R,
				Ampicillin + Sulbactam-R, Piperacillin-R
			*P. vulgaris*	Ampicillin-R, Amoxicillin-R,
				Piperacillin-R, Imipenem-R, Moxifloxacin-R
			*S. aureus*	Penicillin-R
17	swab/ cervical	6	*E. coli* ^1^ *E. faecalis*	S S
23	swab/ cervical	7	*K. oxytoca* ^1^	Ampicillin-R, Amoxicillin-R, Piperacillin-R
28	swab/ cervical	3	*E. coli* ^1^	S
29	swab/ cervical	5	*K. oxytoca* ^1^	Ampicillin-R, Amoxicillin-R, Piperacillin-R

R = resistant; S = sensitive to all antibiotics tested; ^1^ species of the *Enterobacteriaceae.*

## Data Availability

The data sets used and/or analyzed during the current study are available from the corresponding author on reasonable request.
